# CDK3, target of miR-4469, suppresses breast cancer metastasis *via* inhibiting Wnt/β-catenin pathway

**DOI:** 10.18632/oncotarget.18171

**Published:** 2017-05-25

**Authors:** Ting Cao, Tian Xiao, Guanqun Huang, Yafei Xu, Joe Jiang Zhu, Kaixin Wang, Wencai Ye, Hong Guan, Jinsong He, Duo Zheng

**Affiliations:** ^1^ Department of Cell Biology and Genetics, Shenzhen University Health Science Center, Shenzhen 518060, Guangdong Province, China; ^2^ Department of Breast Surgery, The First Affiliated Hospital of Shenzhen University, Second People’s Hospital of Shenzhen, Shenzhen 518035, Guangdong Province, China; ^3^ Department of Pathology, The First Affiliated Hospital of Shenzhen University, Second People’s Hospital of Shenzhen, Shenzhen 518035, Guangdong Province, China; ^4^ Guangdong Province Key Laboratory of Pharmacodynamic Constituents of TCM and New Drugs Research, Jinan University, Guangzhou 510632, Guangdong Province, China; ^5^ Department of Pathology, Shenzhen Nanshan People’s Hospital, Shenzhen 518052, Guangdong Province, China

**Keywords:** CDK3, breast cancer, metastasis, microRNA, Wnt pathway

## Abstract

Cyclin-dependent kinase 3 (CDK3), a member of CDK family, is involved in G_0_/G_1_ and G_1_/S cell cycle transitions. Although several researchers discovered that CDK3 related to cell growth in some kinds of cancer, the functions of CDK3 during tumor development remains unclear. Here, we first found that the expression of CDK3 was higher in primary tumors of non-metastatic breast cancer compared with those in metastatic breast cancer. Overexpression of CDK3 suppressed cell migration and invasion of breast cancer cells, and decreased the metastasis in nude mice. We further identified miR-4469 was a negative regulator of CDK3 by directly targeting its 3′-untranslated region (UTR). The increase of motility induced by miR-4469 could be abolished by CDK3 overexpression. Moreover, RNA-seq analysis revealed that Wnt pathway may be inhibited by CDK3 expression, which was subsequently confirmed by western blot. Moreover, Wnt3a treatment abolished the inhibitory role of CDK3 in cell motility, suggesting that Wnt signaling is the potential downstream of CDK3. In conclusion, these results support that CDK3 which is targeted by miR-4469 suppresses breast cancer metastasis by inhibiting Wnt/β-catenin pathway.

## INTRODUCTION

Cyclin-dependent kinases play the essential roles in control of cell cycle progression by interacting with a variety of regulators and substrates [1]. Mutations, abnormal expression and dysfunction of CDKs lead to loss of control on cell proliferation, instability of genome, tumor genesis and tumor progression, which are closely related to breast cancer, lung carcinoma, melanoma, lymphoma, *et cetera* [2–4]. CDK3 is one important member of CDKs family, which is reported to be critical for cell cycle exiting from G_0_ phase and G_1_/S transition [5, 6]. According to the present literatures, CDK3 could enhance Myc-induced proliferation and anchorage-independent growth in Ratl cells [7]. CDK3 also promotes proliferation and transformation of mouse epidermal JB6 cells through up regulating the phosphorylation level of ATF1 [8]. Moreover, CDK3 increases AP-1 transactivation resulted in an increase of Ras-induced transformation in NIH3T3 cells [9], and promotes skin cancer cell growth *via* elevating the phosphorylation level of its binding transcriptional factor NFAT3 [10]. These findings suggested that CDK3 could act as a tumor promoter, due to its ability of promoting cell growth and transformation.

MicroRNAs, which are approximately 21-nucleotide-long noncoding RNA, anneal in the 3′-UTR of protein-coding mRNAs leading to repression of translational efficiency and/or decreased mRNA levels [11, 12]. MiRNAs can function as oncogenes or tumor suppressor genes depending on their gene targets [13, 14]. Analysis of human breast tumors revealed a lot of miRNAs were dysregulated and involved in post-transcriptional regulation [15]. With the development of deep sequencing approach, a growing number of new miRNAs have been identified [16, 17]. However, due to the rare expression of some predicted new miRNAs in tissues, some researchers doubt the real existence of these miRNAs, and there is almost no functional study on them in literatures. Here, according to bioinformatic prediction, we found that miR-4469 is a potential regulator of CDK3. MiR-4469 is firstly reported as a novel miRNA identified by sequencing in malignant human B cells [18], then it is reproducibly detected in paired normal and tumor breast tissue [19], though there is no further study of its roles in cancer. Interestingly, the functions of other newly discovered miRNAs from the same reference have been investigated. For instance, it is verified that miR-4728 could act as a marker of HER2 status in breast cancer [20]; miR-4661 targeting IL-10 influences autoimmune and inflammatory diseases [21]; miR-4723 inhibits prostate cancer growth through inactivation of c-Abl [22]. Thus, we assume that miR-4469 is an existing miRNA and its role in cancer should be elucidated.

In this study, we demonstrated that CDK3 is highly expressed in primary tumors of non-metastatic breast cancer compared with those in metastatic breast cancer and CDK3 suppresses breast cancer metastasis. MiR-4469 could directly target CDK3 and reduce the protein level of CDK3. We further revealed that Wnt/β-catenin signaling pathway is involved in CDK3-mediated regulation of cell motility. Taken together, these data suggested that CDK3, which is targeted by miR-4469, plays an inhibitory role in breast cancer metastasis by inhibiting Wnt/β-catenin pathway.

## RESULTS

### CDK3 expression negatively correlates with metastasis in breast cancer

**Figure 1:**
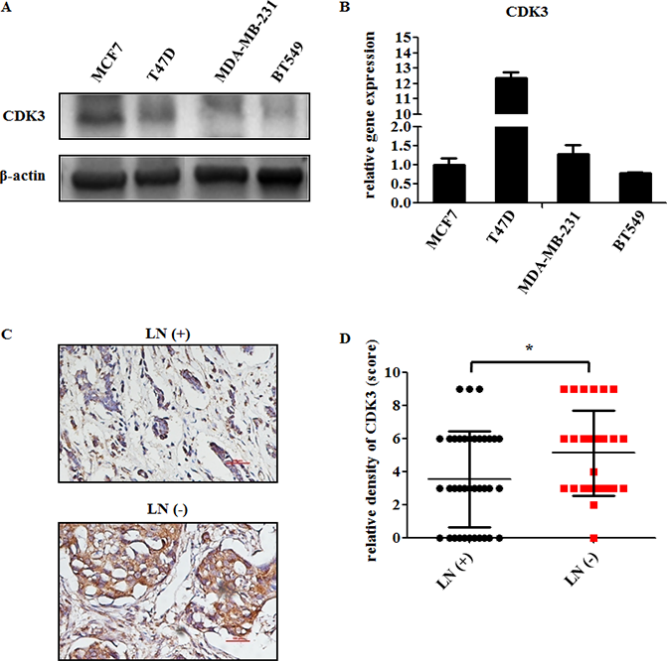
CDK3 is highly expressed in non-malignant breast cancer (**A**) CDK3 expression was detected by western blot in both non-malignant breast cancer cell lines MCF7, T47D, and malignant breast cancer cell lines MDA-MB-231, BT549. β-Actin was used as a loading control. (**B**) The mRNA level of CDK3 in these cell lines was analyzed by quantitative PCR (qPCR). The relative gene expression was normalized by CDK3 level in MCF7 cells. (**C**) Representative immunohistochemical analysis of a breast cancer sample from a patient with lymph node metastasis [LN(+)], or without lymph node metastasis [LN(-)], labeled by CDK3 antibody (magnification, ×200). (**D**) CDK3 staining was evaluated and used for statistical analysis (* *P* < 0.05).

To investigate the potential role of CDK3 in breast cancer, we first examined CDK3 expression in different breast cancer cell lines. The protein level of CDK3 was higher in non-malignant cancer cell lines (MCF7, T47D), compared with malignant cancer cell lines (MDA-MB-231, BT549) (Figure 1A). However, CDK3 mRNA level was not consistent with the protein level, suggesting that the expression of CDK3 was affected by post-transcriptional regulation (Figure 1B). Moreover, to further determine the relationship between CDK3 and breast cancer metastasis, CDK3 expression was detected by immunohistochemisty in formalin-fixed and paraffin-embedded clinical tissues, including 37 cases of lymph node metastatic breast cancer tissues, and 28 cases of lymph node non-metastatic breast cancer tissues (Figure 1C). The detailed clinical information of tissue samples has been listed in Supplementary Table 1. Meanwhile, CDK3 staining scores were evaluated according to staining intensity and proportion of positive stain (Figure 1D). The immunohistochemistry results revealed that CDK3 was highly expressed in primary tumor tissues of non-metastatic breast cancer, implying that CDK3 might be involved in breast cancer metastasis. In addition, we examined CDK3 expression by immunohistochemisty in normal breast tissue and breast cancer tissue by using a tissue microarray including 59 cases of normal breast tissues and 194 cases of breast cancer tissues (Supplementary Figure 1A and 1B). This result showed that CDK3 exhibited a lower level in normal breast tissues than breast cancer tissues.

### CDK3 suppresses the motility of breast cancer cells

**Figure 2:**
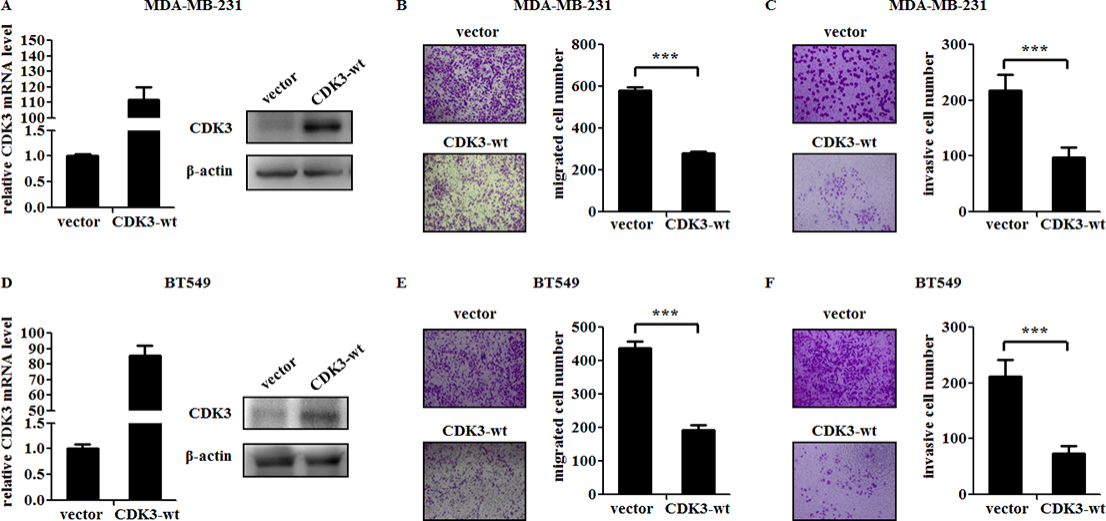
CDK3 overexpression suppresses the motility of breast cancer cells (**A**) and (**D**) Overexpression of CDK3 in MDA-MB-231 or BT549 cells were detected by qPCR (left) and western blot (right). Migration of MDA-MB-231 or BT549 cells were shown in (**B**) and (**E**). Representative photos were shown in left, and quantified data was shown in right. Invasion of MDA-MB-231 or BT549 cells were shown in (**C**) and (**F**). Representative photos were shown in left, and quantified data was shown in right (***P < 0.001).

**Figure 3:**
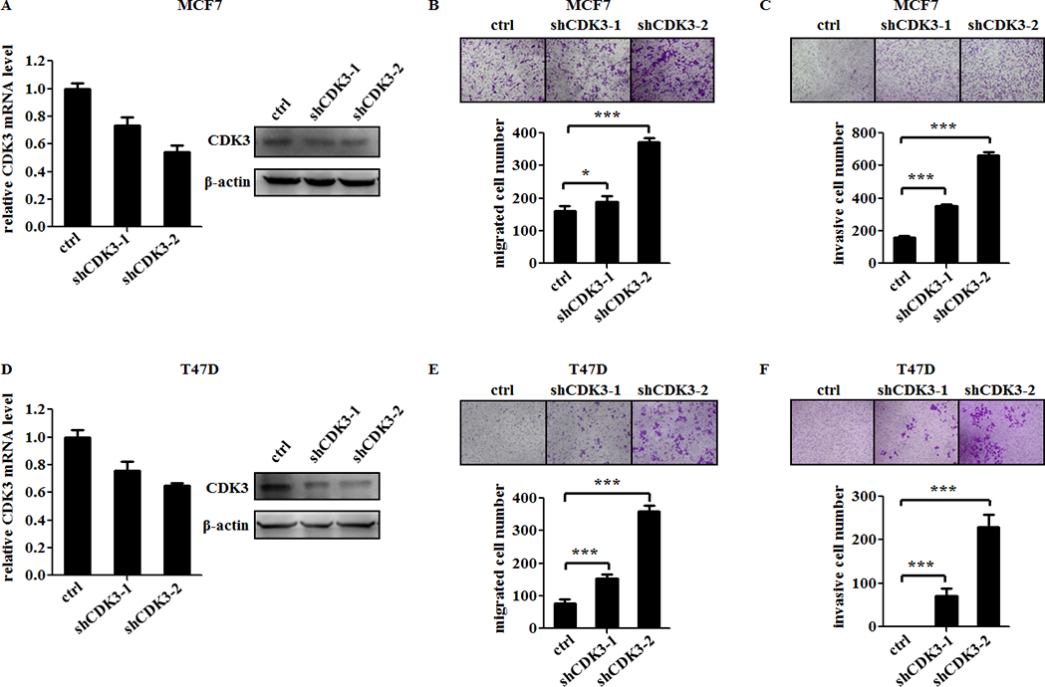
CDK3 knockdown increases the motility of breast cancer cells (**A**) and (**D**) Knockdown efficiency of CDK3 in MCF7 or T47D cells were detected by qPCR (left) and western blot (right). Migration of MCF7 or T47D cells were shown in (**B**) and (**E**). Representative photos were shown in left, and quantified data was shown in right. Invasion of MCF7 or T47D cells were shown in (**C**) and (**F**). Representative photos were shown in left, and quantified data was shown in right (*P < 0.05, ***P < 0.001).

To further investigate the role of CDK3 in breast cancer metastasis, we infected malignant breast cancer cell line MDA-MB-231 and BT549 cells with lentivirus-expressing wild-type CDK3. The expression of CDK3 was up-regulated at both mRNA level and protein levels (Figure 2A and 2D). Meanwhile, the motility and invasive ability of MDA-MB-231 and BT549 cells were both significantly reduced assessed by Boyden chamber assay (Figure 2B, 2C and 2E, 2F). On the other hand, knockdown of CDK3 *via* two different short hairpin RNAs (shRNAs) in non-malignant breast cancer cell line MCF7 and T47D strongly reduced endogenous CDK3 expression at both mRNA and protein levels (Figure 3A and 3D), and subsequently increased cell motility and invasive ability (Figure 3B, 3C and 3E, 3F), further indicating that CDK3 plays an inhibitory role in breast cancer metastasis. Furthermore, we examined cell proliferation in CDK3-overexpressed MDA-MB-231 cells and CDK3-silenced MCF7 cells. Interestingly, altered the expression of CDK3 had no effect on cell proliferation (Supplementary Figure 2A and 2B). However, CDK3 overexpression dramatically decreased colony formation of MDA-MB-231 cells in 2-D culture dishes (Supplementary Figure 2C), suggesting that CDK3 is also related with clonogenic ability of breast cancer cells.

### Overexpressed CDK3 in MDA-MB-231 cells suppresses metastatic dissemination

**Figure 4:**
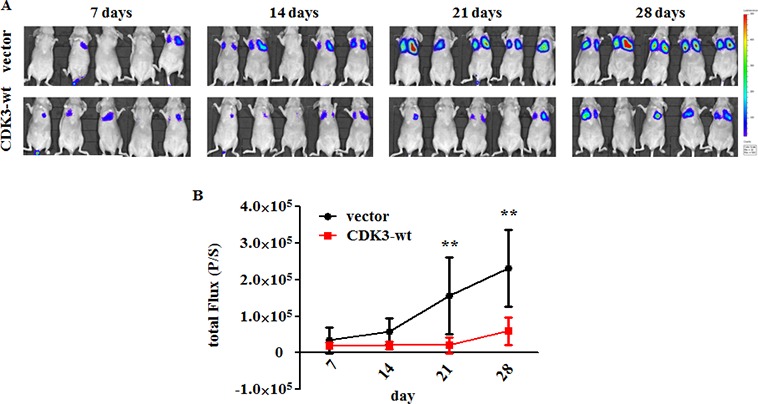
CDK3 inhibits breast cancer metastasis *in vivo* (**A**) Bioluminescent images of BALB/c nude mice were measured every 7 days after i.v. injection. False color logarithmic scale represents the intensity of the bioluminescent signal in photons. (**B**) Quantification of the total flux of the bioluminescent signal per mouse in photons per second over the course of the experiment. Values represent the average of the total flux for all mice on the indicated day. *P*-values were calculated using the Mann-Whitney *t*-test (**P < 0.01).

We further used an experimental metastasis mouse model to confirm the function of CDK3 *in vivo*. We performed tail-vein injection of MDA-MB-231-luci-CDK3-wt and control cell lines in nude mice. As determined by total body luminescence, MDA-MB-231-luci-CDK3-wt exhibited a dramatic inhibition of experimental metastasis compared to the control cells (Figure 4A and 4B), supporting the suppressive role of CDK3 in breast cancer metastasis.

### MiR-4469 promotes breast cancer cell motility by targeting CDK3

**Figure 5:**
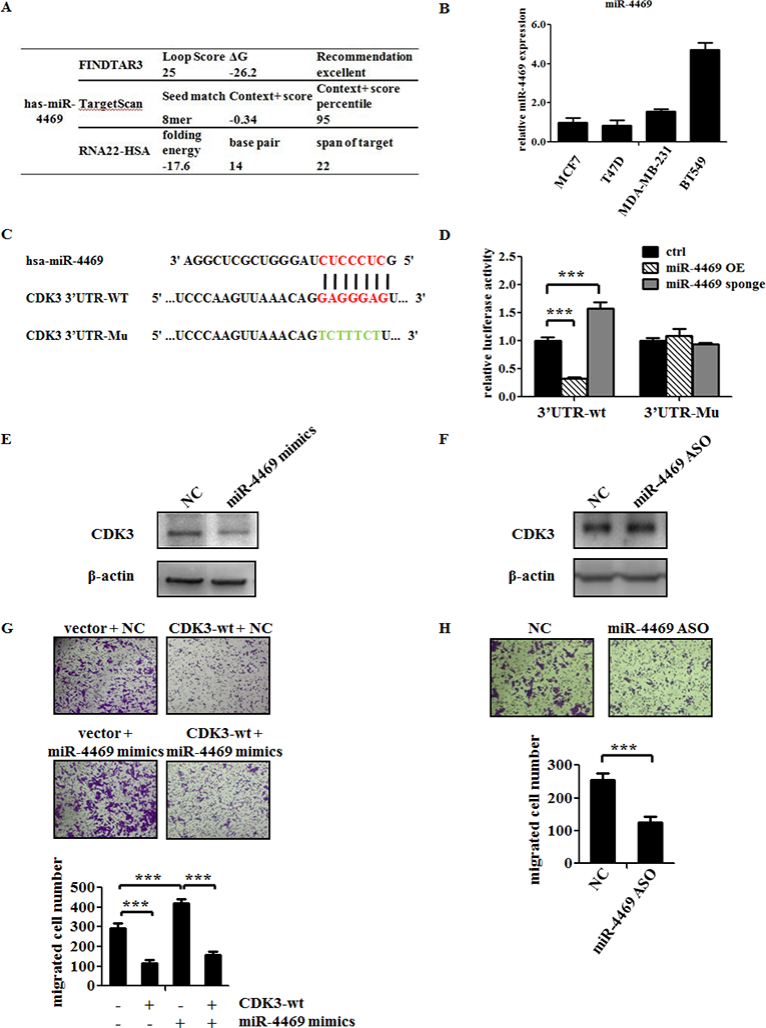
MiR-4469 targets CDK3 3′UTR and promotes the motility of breast cancer cell (**A**) Bioinformatics prediction of the bind possibility between miR-4469 and CDK3 3′UTR. (**B**) Real-time PCR analysis of miR-4469 levels in MCF7, T47D, MDA-MB-231 and BT549 cells. (**C**) Gene structure of CDK3 showing the predicted target site of miR-4469 in its 3′UTR. (**D**) HEK293T cells were transfected with reporter gene containing wild-type (3′UTR-wt) or mutant CDK3 3′UTR (3′UTR-Mu) along with miR-4469 OE (overexpression) or miR-4469 sponge plasmid (***P < 0.001). (**E**) and (**F**) Western blot analysis of CDK3 protein level in MCF7 cells transfected with miR-4469 mimics or miR-4469 ASO. (**G**) Transwell assay was performed in MCF7 cells transfected with CDK3-wt or co-transfected with CDK3-wt and miR-4469 mimics, and quantified data was shown below. (**H**) Transwell assay in MCF7 cells transfected with negative controlor miR-4469 ASO, and quantified data was shown below (***P < 0.001).

As shown in Figure 1, the incompatible expression of CDK3 mRNA level and protein level is maybe due to the post-transcriptional regulation. Thus, we speculated some miRNAs may mediate this regulation. To identify the miRNAs targeting CDK3, several computational methods were used. Among those potential miRNAs, miR-4469 is of particular interest due to its high predicted score in all three online bioinformatics methods (Figure 5A).

Therefore, a series of experiments were carried out to determine whether CDK3 is the direct target of miR-4469. First, the expression of miR-4469 was assessed by qPCR among breast cancer cell lines. Consistent with our hypothesis, the level of miR-4469 was abundant in malignant breast cancer cell lines, which is in contrary to the protein level of CDK3 (Figure 5B). Further, we generated plasmids containing wild-type or mutant CDK3-3′ UTR that were fused to a luciferase reporter according to the binding sites between CDK3-3’UTR and miR-4469 (Figure 5C). Dual luciferase assay showed that miR-4469 overexpression significantly inhibited luciferase activity of wild-type but not mutant reporter genes in 293T cells, while miR-4469 silencing specifically enhanced the luciferase activity of wild-type but not mutant CDK3-3′UTR (Figure 5D). Although altering the expression of miR-4469 did not change CDK3 mRNA levels (data not shown), overexpression of miR-4469 indeed resulted a decrease of CDK3 protein level (Figure 5E). However, silencing of miR-4469 caused an increase in CDK3 protein level in MCF7 cell (Figure 5F). Thus, these findings suggest that miR-4469 targets CDK3 through translational inhibition.

Then, we explored if miR-4469 could enhance breast cancer cell motility through targeting CDK3. Up-regulation of miR-4469 promoted MCF7 cell motility, however, overexpression of CDK3 could abolish the increased motility caused by miR-4469 (Figure 5G). In contrary, silencing of miR-4469 by using its anti-sense, resulted in a reduction of MCF7 cell motility (Figure 5H). Altogether, these findings demonstrate that CDK3 is the direct target of miR-4469 and miR-4469 could increase the motility of breast cancer cells through targeting CDK3.

### CDK3 inhibits Wnt/β-catenin signaling pathway in breast cancer

To elucidate the mechanism of suppression of cell motility and metastasis by CDK3 in breast cancer, we examined the mRNA expression profile in both CDK3-overexpressed MDA-MB-231 or BT549 cells, and CDK3-silenced MCF7 cells. The sheet of genes with great changes (logFC>2, or logFC<-2) in each set was shown in Supplementary Table 2. Then, we performed Gene Ontology analysis of the three sets of genes. More concretely, top 50 down-regulated genes in CDK3 overexpressed BT549 cells were used for analyzing *via* PANTHER Classification System (http://pantherdb.org/), and suggestive pathways were given that the genes participate in. Top 50 up-regulated genes in CDK3 silenced MCF7 cells were analyzed in the same way. Since in CDK3-overexpressed MDA-MB-231 cells, there were only 37 genes with logFC<-2, they were used for analysis in this set. Another analysis using top 50 up-regulated genes in BT549 and MDA-MBA-231 sets, top 50 down-regulated genes in MCF7 set was shown in Supplementary Figure 3.

**Figure 6:**
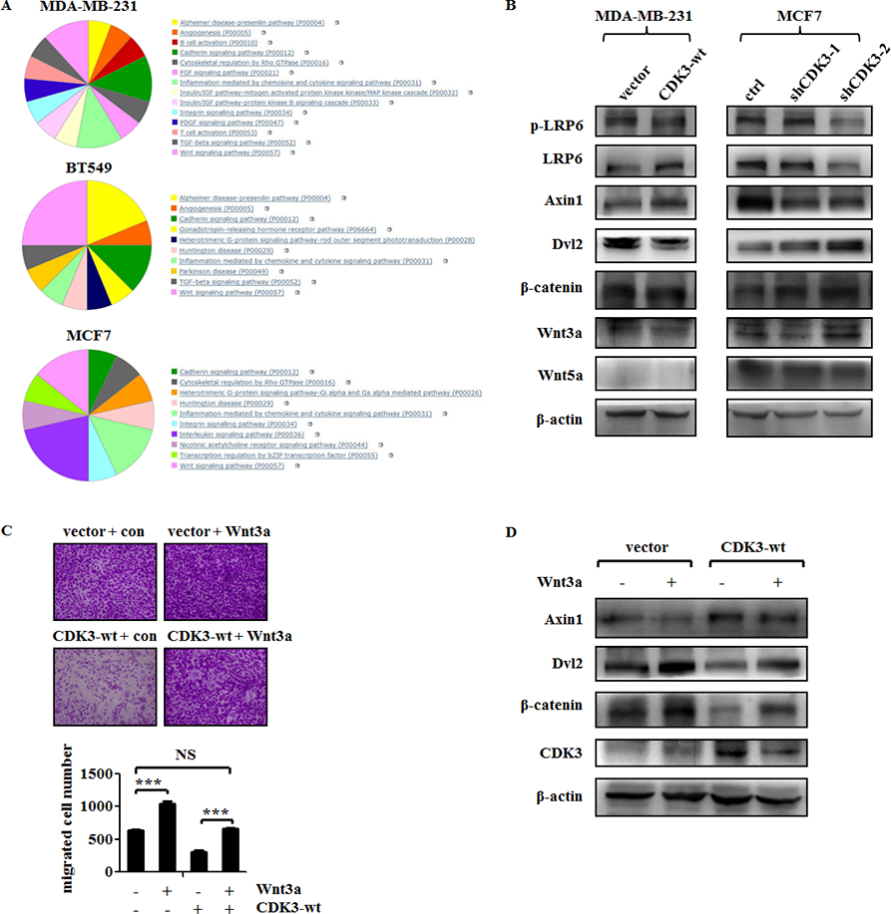
CDK3 inhibits Wnt/β-catenin signaling in breast cancer cells (**A**) Gene ontology analysis of top 37 genes that had 2-fold decrease in MDA-MB-231 cells overexpressed CDK3, top 50 genes had 2-fold decrease in BT549 cells overexpressed CDK3, and top 50 genes had 2-fold increase in MCF7 cells silenced CDK3. (**B**) Western blot was performed to detect molecules expression involved in Wnt/β-catenin signaling in both CDK3 overexpressed MDA-MB-231 cells and CDK3 silenced MCF7 cells. (**C**) Transwell assay was performed in Wnt3a conditional medium or control medium treated MDA-MB-231 cells, and quantified data was shown below. (**D**) Western blot was performed to detect Wnt/β-catenin signaling in Wnt3a treatment (***P < 0.001, NS indicates not significant).

Among these showed pathways, there are only three pathways appeared in all three sets: cadherin signaling pathway, inflammation mediated by chemokine and cytokine signaling pathway, and Wnt signaling pathway (Figure 6A). Since inflammation pathway is complex and easily influenced by cytokines, and cadherin pathway is considered as the downstream of Wnt signaling [23], we concerned if Wnt signaling pathway is regulated by CDK3. To verify this, western blot for detecting molecules related to Wnt signaling was performed. First, we noticed that CDK3 overexpression decreased Wnt3a protein level, while Wnt5a was not affected, suggesting that Wnt canonical pathway was involved in CDK3 regulation. Moreover, we explored other signals in this pathway. As shown in Figure 6B, CDK3 overexpression decreased the phosphorylation level of LRP6, which could form the receptor complex with Frizzled, followed by decreased level of Dvl2 and increased level of Axin1, and ultimately decreased the level of β-catenin. However, in CDK3-silcenced MCF7 cells, the phosphorylation level of LRP6 was not significantly affected by CDK3, but Dvl2 was up-regulated, resulted in a decreased level of Axin1 and increased level of β-catenin.

To further investigate the role of Wnt signaling in CDK3-associated cell motility, we treated CDK3-overexpressed MDA-MB-231 cells with Wnt3a conditional medium. As demonstrated in Figure 2B and 2C, up-regulation of CDK3 could inhibit MDA-MB-231 cell motility, whereas treatment of Wnt3a abolished the inhibitory role of CDK3 in cell migration (Figure 6C). Western blot results further showed that treatment of Wnt3a conditional medium dramatically increased expression of β-catenin and Dvl2 but decreased Axin1 level, demonstrating that Wnt/β-catenin signaling pathway was re-activated (Figure 6D). In conclusion, our data identified CDK3 as a suppressor of metastasis in breast cancer and indicated that Wnt/β-catenin signaling was the potential downstream of CDK3.

## DISCUSSION

According to the present literatures, the major function of CDK3 is a promoter for cancer cell growth and transformation by phosphorylating its substrates, such as ATF1, c-Jun, NFAT3 and IK3-1/Cables [8–10, 24]. However, in our study, overexpression of CDK3 exerts an inhibitory effect of breast cancer motility both *in vitro* and *in vivo*, which provides a novel role of CDK3 in cancer.

Higher expression of CDK3 was reported in other cancers, such as glioblastoma [8], skin cancer [10] and colorectal cancer [25]. In our study, we compared CDK3 expression in normal breast tissue and breast cancer tissue by using a tissue microarray. We found that CDK3 exhibited a lower level in normal breast tissues than breast cancer tissues (Supplementary Figure 1). This result was also observed in a previous study on breast cancer, in which the researchers found that CDK3 was overexpressed in breast cancer and phosphorylate ERα at Ser104/116 and Ser118 [26]. In that study, they also demonstrated that CDK3 is required for breast cancer cell proliferation. However, CDK3 protein level and functions were only measured in ER-positive cell lines. It did not mention if CDK3 exerts the similar role in ER-negative tumor cells. Thus, the function of CDK3 on cell proliferation should be further confirmed, especially between ER-positive and ER-negative (e.g. MDA-MB-231) breast cancer cell lines.

As a mediator of cell cycle, CDK3 indeed participate in several tumor cell growth, however, we should not constrain its function only in cell cycle. As Micalizzi.*et al.* described, TGF-β signaling plays a complex role in tumor progression, suppressing tumor formation in normal tissue and early lesions while promoting invasion and metastatic dissemination in later stages of tumor development [27]. Thus, we assume that CDK3 could exhibit different roles in different tumor stages and tumor types, and further experiments need to be performed to verify this hypothesis.

In this paper, we also searched for possible mechanisms for CDK3 regulation. According to the computational methods, miR-4469 was found as a negative regulator of CDK3 by directly targeting CDK3 3′UTR, and subsequent experiments suggested that the up-regulation of miR-4469 level could be responsible for the increase of breast cancer motility. This is also the first time that the function of miR-4469 in cancer is investigated.

Moreover, the signaling pathway induced by CDK3 was estimated based on RNA-seq results among breast cancer cell lines altered the expression of CDK3. We observed that Wnt signaling pathway related genes had predominant changes in all three tested groups, and the changes were further confirmed by western blot. The Wnt signaling pathway plays a significant role in physiology and pathology of breast [23, 28, 29]. As shown here, CDK3 exhibited a potent inhibitory effect on Wnt signaling in breast cancer cells through regulating the phosphorylation level of LRP6, the expression of Axin1 and Dvl2, leading to the inhibition of β-catenin, and this process is possibly due to decreased expression of Wnt3a. However, the level of Wnt5a was not affected by CDK3. Since Wnt3a is the major ligand for Frizzled mediated Wnt canonical pathway [30]. It is strongly providing the evidence that CDK3 exerts the inhibitory role in breast cancer motility by inhibiting Wnt/β-catenin pathway.

## MATERIALS AND METHODS

### Cell culture

HEK293T cell line and the human breast cancer cell lines MCF7, T47D, MDA-MB-231 and BT549 were purchased from American type culture collection (ATCC, Manassas, VA, USA). Cell lines were authenticated on the basis of viability, recovery, growth and morphology. All cells were cultured in Dulbecco’s modified Eagle’s media medium containing 10% fetal bovine serum (Hyclone, Thermo Fisher Scientific, Florence, KY, USA) at 37°C with 5% CO2 in a tissue culture incubator. These cells are regularly tested to ensure that they are mycoplasma free.

### Plasmids and lentiviral infection

Full-length human CDK3 cDNA was amplified from a human mRNA pool generated by RT-PCR using SuperScript II RNase H Reverse Transcriptase (Life Technologies), and the PCR products was then cloned into pCDH-CMV (System Biosciences, Mountain View, CA, USA). CDK3 shRNA oligos were synthesized by Life Technology and cloned into the pLKO.1 expression construct (using pLKO.1-scramble shRNA as control). High-titer lentivirus was generated by transient transfection of HEK293T cells. Cells were then infected with the viral supernatant fractions supplemented with polybrene. The culture medium was replaced with fresh growth medium with puromycin for selection at 16 h post-infection. The cells were cultured in selected medium until control cells completely died and the overexpression and knockdown efficiency were then evaluated by qPCR and western blot. MiR-4469 mimics and anti-sense oligonucleotides were purchased from GenePharma (Shanghai, China). MiR-4469 overexpression plasmid and miR-4469 sponge plasmid were purchased from Genechem (Shanghai, China).

### Real-time PCR

Total RNA was isolated from cells with Trizol (Invitrogen) reagent, according to the manufacturer’s instructions. MicroRNA was extracted by using Buffer MZ (TIANGEN, Beijing, China). MicroRNA from each sample was reverse transcribed with miRcute Plus miRNA First-Strand cDNA Synthesis Kit (KR211, TIANGEN, Beijing, China). Real-time PCR was performed with SYBR Green (Roche) detecting by Bio-Rad CFX connect Real-time System (Bio-Rad, USA). The expression level of mature miRNAs was calculated using U6 as an internal control.

### Western blot

Protein concentration was determined using BCA Protein Assay Kit (Thermo, USA). For western blots, CDK3 antibody was obtained from Abcam (ab96847), p-LRP6, LRP6, Axin1, Dvl2, β-catenin, Wnt3a and Wnt5a antibodies were purchased from Cell Signaling (Wnt Signaling Antibody Sampler Kit #2915).

### Immunohistochemistry staining

Immunohistochemistry staining of human breast cancer tissues was carried out essentially as previously described [31]. In brief, the slide was first deparaffinized and subjected to heat-induced antigen retrieval using citrate buffer. The tissue sections were then incubated with primary antibodies against CDK3 (sc-826, Santa Cruz) overnight at 4°C, followed by extensive washes. The slide was subsequently treated with biotinylated anti-rabbit secondary antibody and then developed using avidin-conjugated horseradish peroxidase with diaminobenzidine as the substrate. Hematoxylin was used for counterstaining and the images were visualized and scored under microscope. Human breast cancer tissue slices were obtained from the First Affiliated Hospital of Shenzhen University with the informed consent of patients and with approval for experiments from the First Affiliated Hospital of Shenzhen University.

### Transwell assay

5 × 10^4^ cells were seeded in 200 μl of serum-free medium onto the upper chamber of 24-well transwell inserts (8-μm pores, Corning, Thermo Fisher, USA) coated with or without matrigel (BD Biosciences, USA). The lower chamber contained 600 μl of DMEM medium with 10% FBS. After incubation at 37°C for 16 h (MDA-MB-231 and BT549 cells) or 24 h (MCF7 and T47D cells), the non-invaded cells on the upper side of membrane were removed by a cotton swab, and the migrated or invasive cells were stained with 2% crystal violet and then observed under the microscope.Wnt3a conditional medium and control medium were harvested from Wnt3a expressing plasmid transfected HEK293T cells after 48 hours, and treated MDA-MB-231 cells for 24 hours before motility experiment. All the results of transwell assay have been quantified by counting the migrated or invasive cell number.

### *In vivo* experiments

Female BALB/c nude mice (5–7 weeks old) were purchased from Beijing Vital River Laboratory Animal Technology Company and maintained and treated under specific pathogen-free conditions. Breast cancer cells MDA-MB-231-luci (2 × 10^6^ per mouse) were injected i.v. *via* the tail vein. The mice were measured for their bioluminescent signal approximately every 7 days. Values represent the average bioluminescent signal per mouse in photons/second. All the experimental procedures involving animals were conducted in accordance with protocols approved ethically by the Institutional Animal Use Committee of the Health Science Center, Shenzhen University.

### Prediction of miRNA targeting to CDK3

The miRNA databases and target prediction tools TargetScan (http://www.targetscan.org/vert_61/), FINDTAR3 (http://bio.sz.tsinghua.edu.cn/) and RNA22-HSA (https://cm.jefferson.edu/rna22/Precomputed/) were used to identify potential miRNA binding to CDK3 3′UTR.

### 3′UTR-luciferase reporter gene assay

The wild-type or mutant 3′UTR of CDK3 containing the predicted miR-4469 binding sites was cloned into the pRL-TK vectors. 100 ng of pRL-TK-3′UTR-wt or mutant and 20 ng of pGL3.0 control vector were co-transfected in 293T cells planted in 24 well plate. Four hours after this transfection, 0.5 ug of miR-4469 overexpression plasmid or miR-4469 sponge plasmid was transfected into the cells respectively. After another 48 h, cells were lysed, and luciferase activities were measured using dual luciferase assay kit (Promega).

### RNA-Seq analysis

RNA-Seq was carried out essentially as previously described [32]. In brief, total RNA was isolated by homogenization in 1 ml of Trizol reagent. Total RNA concentration, 260/230 and 260/280 ratios were estimated by Nanodrop spectrometer N1000 (Thermo Scientific). RNA integrity was estimated by Agilent 2100 bioanalyzer. A total of 3 μg of RNA per sample was used for sequencing. After normalization, differentially expressed mRNA were identified through Fold Change filtering.

### Statistical analysis

For immunohistochemistry staining, the staining intensity and proportion of positive stains were assessed by the following criteria. For the staining intensity, a score of 0 indicates negative staining, 1 indicates weak staining, 2 indicates moderate staining and 3 indicates strong staining. Regarding the proportion of the positive stain area, 0 indicates no staining, 1 indicates 0–20% staining, 2 indicates 21–60% staining and 3 indicates 61–100% staining. The total score was the multiply of the staining intensity score and the proportion of positively stained area. The data was analyzed by the Mann-Whitney nonparametric two-tailed *t*-test.

Experiments were performed in triplicate, unless otherwise stated. Data are expressed as mean value ± S.D. and were analyzed by one-way analysis of variance followed by the Student-Neuman-Keul’s test. *P* values are indicated in the legend of each figure.

## SUPPLEMENTARY MATERIALS FIGURES AND TABLES


