# Supplementary material for: CDK3, target of miR-4469, suppresses breast cancer metastasis via inhibiting Wnt/β-catenin pathway
Source: Oncotarget. 2017 May 25;8(49):84917–27. doi: 10.18632/oncotarget.18171 (PMC5689583; doi:10.18632/oncotarget.18171)
Supplement: Supplementary file 1 [file oncotarget-08-84917-s001.pdf]

CDK3, target of miR-4469, suppresses breast cancer metastasis *via* inhibiting Wnt/β-catenin pathway

Supplementary Materials

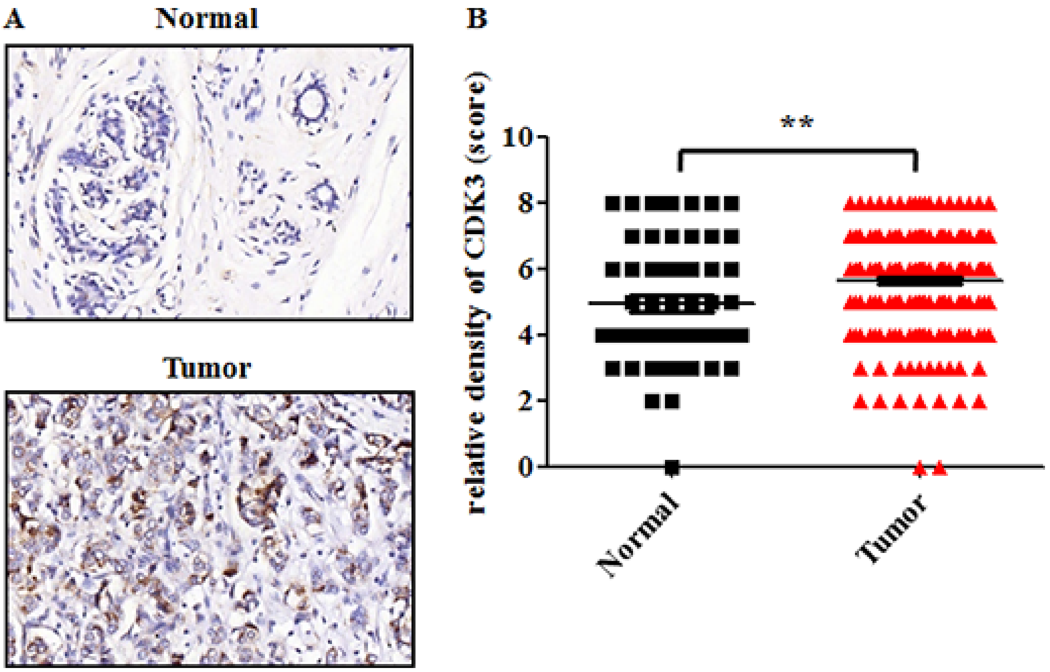

Supplementary Figure 1: CDK3 is highly expressed in breast tumor tissues. (**A**) Representative immunohistochemical analysis of normal breast tissue and tumor labeled by CDK3 antibody (magnification, ×200). (**B**) Evaluation of CDK3 staining by immunohistochemisty assay in 59 cases of normal breast tissues and 194 cases of breast cancer tissues (***P* < 0.01).

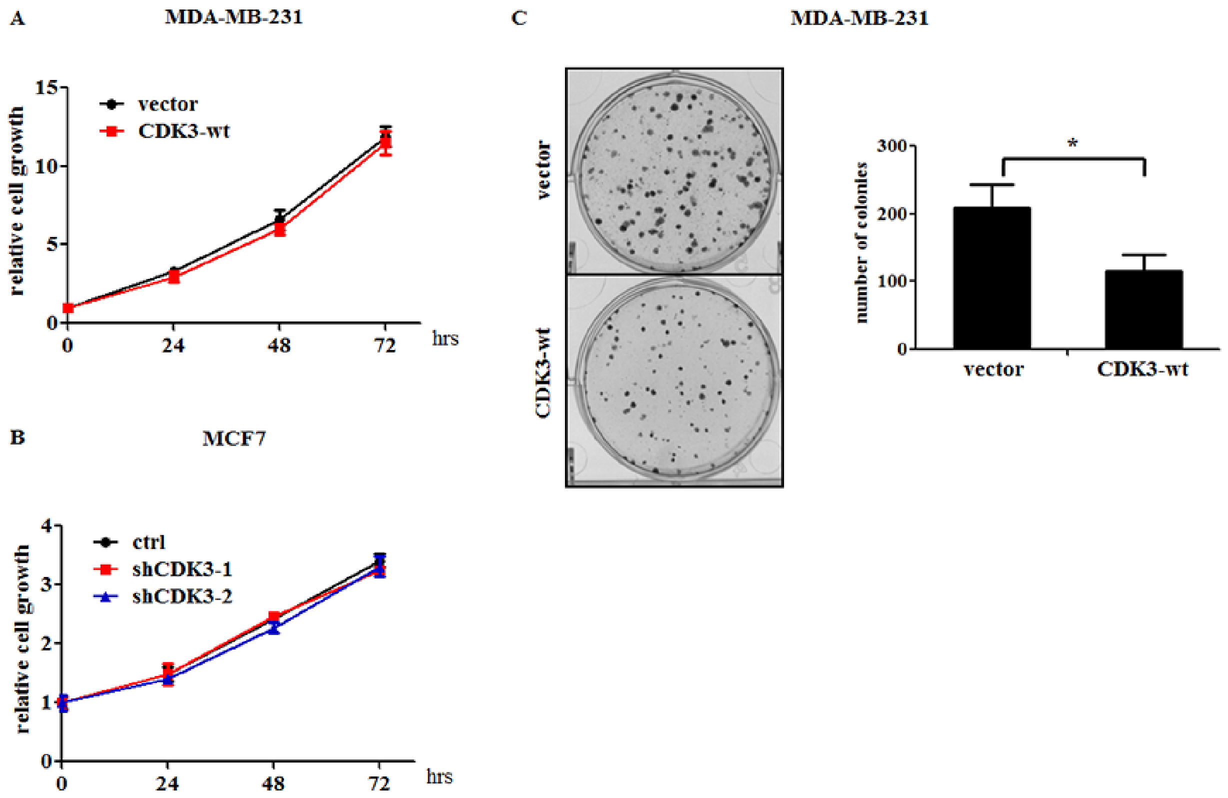

Supplementary Figure 2: The effect of CDK3 expression on breast cancer cell proliferation and clonogenic ability. (**A** and **B**) Proliferation of stable MDA-MB-231 and MCF7 cells was measured by MTS assay. (**C**) Representative photos and statistical analysis of 2-D colony formation in stable MDA-MB-231 cells (*$P < 0.05$).

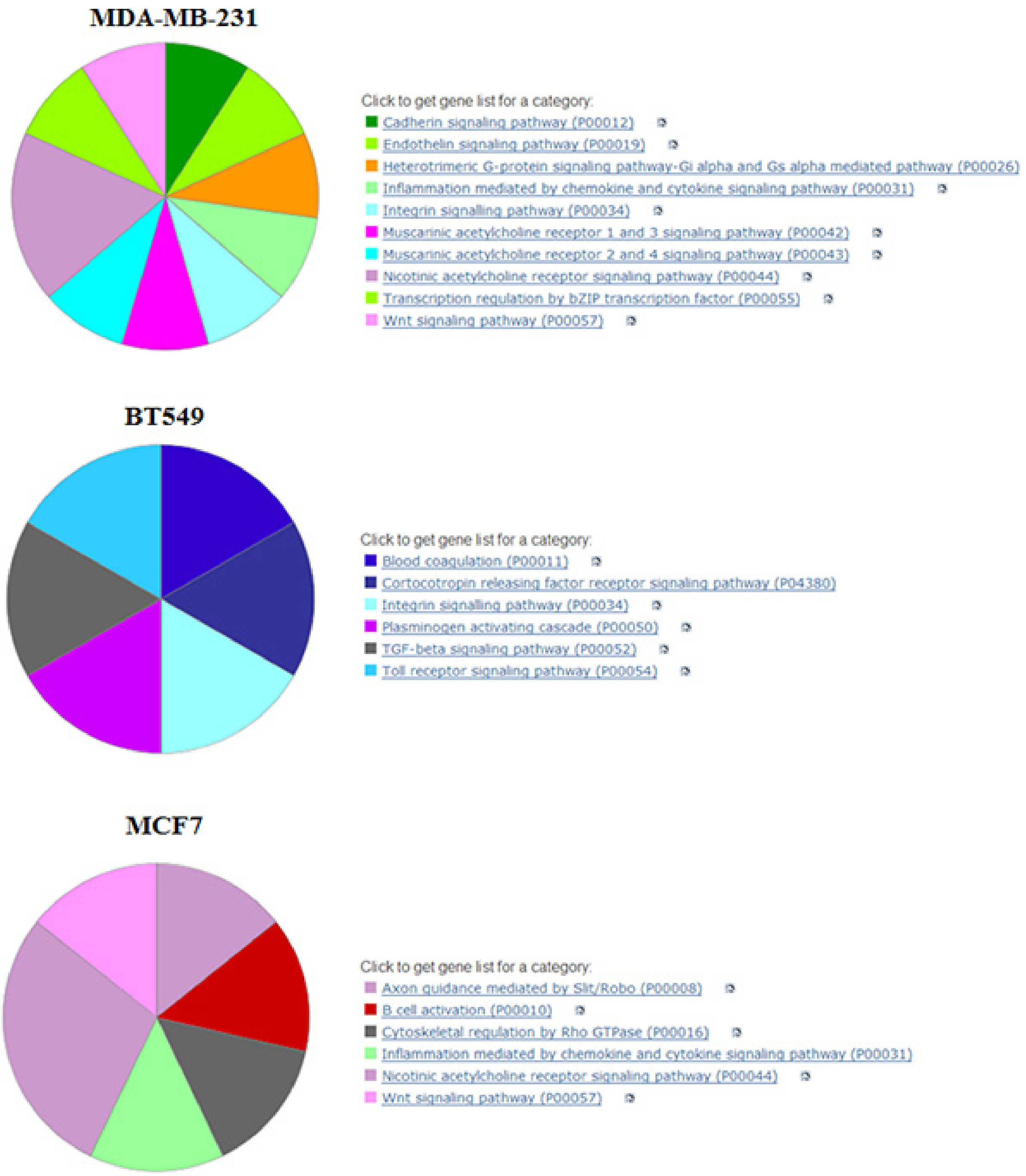

Supplementary Figure 3: Potential signaling pathways regulated by CDK3. Gene ontology analysis of top 50 genes that had 2-fold increase in MDA-MB-231 cells overexpressed CDK3, top 50 genes had 2-fold increase in BT549 cells overexpressed CDK3, and top 50 genes had 2-fold decrease in MCF7 cells silenced CDK3.

Supplementary Table 1: Clinical and pathologic characteristics of 59 breast cancer patients

Pateint No.	Sex	Age	Grade	Stage	TNM	ER	PR	Her-2	LN metastasis
1	Female	43	3	ⅢC	T1N3M0	–	–	+++	17/20
2	Female	36	2	ⅢC	T2N3M0	+	+	+++	13/21
3	Female	67	3	ⅡA	T2N0M0	+++	+	+++	0/18
4	Female	59	1	0	TisN0M0	+	+	+	0/7
5	Female	39	3	ⅡB	T3N0M0	–	–	+	0/22
6	Female	51	2	ⅠA	T1N0M0	+++	–	–	0/26
7	Female	68	2	ⅢC	T2N3M0	+++	+	++	16/24
8	Female	52	2	ⅠA	T1N0M0	–	–	+++	0/20
9	Female	75	2	ⅡA	T2N0M0	+	–	++	0/16
10	Female	47	2	ⅠA	T1N0M0	++	–	+	0/24
11	Female	76	2	ⅢB	T4N1M0	–	–	–	2/30
12	Female	33	2	ⅢC	T2N3M0	++	+	–	5/7
13	Female	58	3	ⅡB	T2N1M0	+++	–	++	2/18
14	Female	64	2	ⅢA	T2N2M0	+++	–	++	4/25
15	Female	55	3	ⅢC	T2N3M0	+	–	+	11/32
16	Female	39	2	ⅡA	T2N0M0	+	+	+	0/24
17	Female	58	3	ⅡA	T2N0M0	–	–	++	0/22
18	Female	81	3	ⅠA	T1N0M0	+++	+	–	0/18
19	Female	39	3	ⅢB	T2N3M0	–	–	++	11/21
20	Female	52	2	ⅠA	T1N0M0	–	–	–	0/19
21	Female	42	3	ⅠA	T1N0M0	–	–	+++	0/19
22	Female	47	3	ⅢA	T3N1M0	++	+	–	1/26
23	Female	35	3	ⅢC	T2N3M0	+	–	+	5/9
24	Female	53	3	ⅡA	T2N0M0	+++	–	+	0/16
25	Female	71	2	ⅠA	T1N0M0	++	–	–	0/26
26	Female	39	3	ⅢA	T3N1M0	++	–	++	2/11
27	Female	39	2	ⅢC	T2N3M0	+++	–	++	35/41
28	Female	60	2	ⅡB	T2N1M0	+++	–	+	1/15
29	Female	70	2	ⅡB	T2N1M0	++	+	–	2/18
30	Female	62	3	ⅡA	T2N0M0	+	+	++	0/25
31	Female	36	3	ⅡA	T2N0M0	+++	+	–	0/32
32	Female	45	3	ⅡA	T2N0M0	+	+	++	0/22
33	Female	46	3	ⅡB	T1N1M0	–	–	–	2/26
34	Female	50	3	ⅢA	T2N2M0	+++	+++	+	3/8
35	Female	52	3	ⅡB	T2N1M0	+	–	++	2/25
36	Female	43	3	ⅡB	T2N1M0	–	–	+++	1/13
37	Female	45	2	ⅢA	T1N2M0	–	–	+++	6/19
38	Female	46	1	ⅡB	T2N1M0	+++	+	++	3/24
39	Female	42	2	ⅡA	T2N0M0	+	–	+	0/18
40	Female	36	2	ⅡA	T2N0M0	+	–	–	0/15
41	Female	47	2	ⅢC	T3N3M0	–	–	+++	18/29
42	Female	50	2	ⅡB	T2N1M0	+	–	+	1/36
43	Female	44	2	ⅢA	T2N2M0	+++	+++	++	7/20
44	Female	45	3	ⅢC	T4N3M0	–	–	+	15/24
45	Female	38	3	ⅢA	T2N2M0	++	+++	++	4/16
46	Female	37	3	ⅠA	T1N0M0	–	–	–	0/21
47	Female	47	2	ⅡA	T2N1M0	+	+	++	1/23
48	Female	41	3	ⅡB	T2N1M0	–	–	+++	2/14
49	Female	64	2	ⅠA	T1N0M0	+	–	–	0/13
50	Female	67	3	ⅡA	T2N0M0	–	–	++	0/28
51	Female	39	3	ⅡA	T2N0M0	–	–	+++	0/26
52	Female	65	1	ⅠA	T1N0M0	+++	+++	–	0/18
53	Female	63	3	ⅡA	T2N1M0	–	–	++	0/16
54	Female	41	2	ⅡB	T2N1M0	+	+	+	2/19
55	Female	51	2	ⅡA	T2N0M0	++	–	+++	0/15
56	Female	50	2	ⅢB	T4N0M0	++	++	++	0/16
57	Female	36	3	ⅢA	T2N2M0	+	–	+++	7/15
58	Female	43	1	ⅠA	T1N0M0	+++	–	–	0/4
59	Female	36	3	ⅡA	T2N0M0	–	–	+++	0/27

Supplementary Table 2: Top 50 genes with great changes (logFC>2, or logFC<-2) in each group

MDA-MB-231 CDK3-wt vs vector				BT5491 CDK3-wt vs vector				MCF7 shRNACDK3-2 vs ctrl			
top 50 genes up		top 50 genes down		top 50 genes up		top 50 genes down		top 50 genes up		top 50 genes down	
**geneID**	**logFC**	**geneID**	**logFC**	**geneID**	**logFC**	**geneID**	**logFC**	**geneID**	**logFC**	**geneID**	**logFC**
CDK3	8.81	PLAC4	–6.92	FBXL13	7.66	INHBB	–8.55	VPS37D	9.32	PDE5A	–10.10
PCDHB7	7.01	CTHRC1	–6.60	GAL3ST1	7.27	ACADL	–7.75	B3GNT6	8.50	KCND3	–8.52
PI16	7.01	DERL3	–6.60	C6	7.11	CNGB1	–7.69	MYOM3	8.40	MYOCD	–8.18
ACHE	6.82	FGF7	–6.60	CSF3	7.11	TCF15	–7.45	ADGRF4	8.36	ISPD	–8.14
CSMD3	6.71	LOC101928105	–6.60	HCG26	7.02	C4orf36	–7.32	SRPX2	8.29	MYH3	–8.14
NLRP7	6.60	VAV1	–6.60	BSPRY	6.93	IGSF10	–7.32	KCNE2	8.21	MLC1	–7.97
OCLM	6.60	ADGRG4	–6.48	KRT15	6.83	EXTL1	–7.24	OXCT2	8.13	MIR17HG	–7.88
SCART1	6.60	C2orf73	–6.48	LOC102723729	6.73	DNAH7	–7.17	IL11	8.13	ZNF25	–7.88
TPI1P2	6.60	ZFP3	–6.48	ALMS1P	6.61	FAIM3	–7.17	RND1	8.09	ADNP-AS1	–7.67
AMT	6.47	C3orf49	–6.34	ETNPPL	6.61	TG	–7.00	LINC00706	8.00	TOPORS-AS1	–7.62
DFNB59	6.47	ID4	–6.34	MMP10	6.61	CELF5	–6.91	C9orf173-AS1	7.95	LOC284581	–7.56
ELMO1	6.47	KLB	–6.34	MSH4	6.61	ACAN	–6.81	CARD16	7.95	PADI2	–7.56
MMRN2	6.47	MYLK3	–6.34	SNAI3	6.61	ACKR2	–6.81	EFHB	7.95	PFN1P2	–7.56
AKR7A3	6.34	SIGLEC16	–6.34	TXK	6.61	HEPH	–6.81	LY6D	7.90	SLIT1	–7.56
ASPRV1	6.34	LRRN4CL	–3.94	CRH	6.49	KLRG1	–6.81	TSPAN5	7.90	ADAM1A	–7.50
C2orf66	6.34	ACTN2	–3.76	CRLF2	6.49	CELF4	–6.70	TBXAS1	7.80	LOC100130950	–7.50
CLEC18B	6.34	LOC101926975	–3.76	SLC14A2	6.49	CCL7	–6.59	FAM117A	7.74	NKX3-1	–7.50
FITM1	6.34	LINC01366	–3.55	STAB1	6.49	FITM1	–6.59	PSG6	7.74	ERC2	–7.43
LINC00311	6.34	C19orf38	–3.44	TLL2	6.49	HLX-AS1	–6.59	TSPAN15	7.69	MARCH1	–7.43
LINC01569	6.34	LINC00211	–3.44	ALOXE3	6.35	LINGO3	–6.59	MARCH4	7.63	PIWIL3	–7.43
LOC101930452	6.34	TUB	–3.39	ATP8A1	6.35	MDFI	–6.59	SPOCD1	7.63	KIAA1456	–7.37
SLITRK3	6.34	DOCK9-AS2	–3.10	C3orf36	6.35	PROM1	–6.59	C14orf132	7.50	TMED10P1	–7.37
LOC100288846	4.29	SBSPON	–3.02	COL10A1	6.35	SCG5	–6.59	GPR87	7.50	C21orf140	–7.30
MST1P2	4.22	RIC3	–3.02	CST6	6.35	CYP7A1	–6.33	S1PR1	5.14	LOC101929099	–7.30
HMGCLL1	4.00	FKBP1B	–2.97	INSM2	6.35	LINC00535	–6.33	SPOCK1	4.90	RHAG	–7.30
PLCG1-AS1	3.74	SGPP2	–2.93	KIAA1683	6.35	LINC01119	–6.33	YPEL4	4.60	LOC100287036	–7.22
MRPL23-AS1	3.64	HESX1	–2.90	LY96	6.35	LINC01393	–6.33	MYH15	4.58	LINC00324	–5.74
ROCK1P1	3.64	B4GALT1-AS1	–2.84	MSANTD1	6.35	MMP12	–6.33	PAEP	4.54	CHN2	–5.62
C3AR1	3.43	GPRC5D	–2.84	CDK3	6.21	NCR3	–6.33	SPINK6	4.35	GPR75	–5.23
PSG4	3.42	IGF2	–2.83	SERPINF2	4.26	SLC27A6	–6.33	IFI16	4.28	NKAPP1	–4.94
SPATA18	3.42	C8orf31	–2.78	FAM183A	4.19	NUDT11	–5.27	PADI3	4.17	CD19	–4.72
NRIP3	3.35	LOC100507547	–2.78	HOXA9	3.76	DCN	–4.28	ZNF516	4.03	FAM83B	–4.72
EPHA5-AS1	3.30	CCNI2	–2.70	DCDC2	3.53	PHYHD1	–3.90	ARNT2	4.00	LOC100190986	–4.46
RHD	3.30	HSP90B2P	–2.62	FGFBP1	3.51	TMEM27	–3.82	FAM83A	3.96	NEXN-AS1	–4.27
FAM184B	3.23	ULK4P3	–2.55	KLHL4	3.47	WNT2	–3.82	C4B	3.91	CCDC144B	–4.17
LINC00685	3.08	PCDHGA11	–2.37	MYOM1	3.47	VCAM1	–3.70	C10orf10	3.86	GCGR	–4.13
TAPT1-AS1	2.91	WNT2B	–2.03	ISM1	3.43	ELN	–3.68	IL6	3.79	PRKXP1	–4.13
ENHO	2.91			DEGS2	3.34	SLC13A5	–3.59	BMF	3.74	EMILIN1	–4.10
ACSBG1	2.82			SLC30A3	3.27	LEF1	–3.48	TGFBI	3.73	PSAPL1	–3.93
ANKRD7	2.81			HEPACAM	3.23	SPOCK3	–3.48	CREB3L1	3.67	POLN	–3.84
TAS2R14	2.76			BEX5	3.03	TLE6	–3.42	CAPN5	3.63	KLRG1	–3.78
TMEM221	2.68			MGC16142	2.99	ZNF521	–3.38	SCN1B	3.53	RGPD1	–3.44
PTPRZ1	2.65			PKP1	2.97	PDZK1	–3.35	PCDH1	3.39	CTAGE9	–3.43
PCBP3	2.60			MAL2	2.87	FAM107A	–3.26	IL4R	3.29	LOC344887	–3.42
CELF2-AS1	2.53			IGFN1	2.85	SEPT7-AS1	–2.94	IL22RA1	3.27	RFXAP	–3.33
CREB3L3	2.48			HOXA10	2.68	COL25A1	–2.85	ACPP	3.23	PAPPA2	–3.20
GUCY1A3	2.35			VNN2	2.63	TIMP3	–2.62	MALL	3.14	KIAA0825	–3.16
NAPSA	2.08			FAM167A	2.63	TTBK1	–2.48	KRT16	3.13	ANO2	–3.11
				IGSF9	2.23	CLUHP3	–2.37	ABCG1	3.13	ZBTB20	–3.08
				PODXL	2.17	COLEC12	–2.31	FAM167A	3.09	LIFR	–2.91
